# Laser induced breakdown spectroscopy for the rapid detection of SARS-CoV-2 immune response in plasma

**DOI:** 10.1038/s41598-022-05509-z

**Published:** 2022-01-31

**Authors:** K. Berlo, W. Xia, F. Zwillich, E. Gibbons, R. Gaudiuso, E. Ewusi-Annan, G. R. Chiklis, N. Melikechi

**Affiliations:** 1 Department of Earth and Planetary Sciences, GEOTOP Research Centre, McGill University, Montreal, Canada; 2 Geriatric Research Education Clinical Center, Bedford VA Healthcare System, Bedford, MA USA; 3 Department of Pharmacology and Experimental Therapeutics, Boston University School of Medicine, Boston, MA USA; 4 Department of Physics and Applied Physics, Kennedy College of Sciences, University of Massachusetts Lowell, Lowell, MA USA; 5 MRN Diagnostics, Franklin, MA USA

**Keywords:** Optics and photonics, Diagnostic markers, Biophysical chemistry, Metals, Viral infection, Mass spectrometry

## Abstract

As the SARS-CoV-2 pandemic persists, methods that can quickly and reliably confirm infection and immune status is extremely urgently and critically needed. In this contribution we show that combining laser induced breakdown spectroscopy (LIBS) with machine learning can distinguish plasma of donors who previously tested positive for SARS-CoV-2 by RT-PCR from those who did not, with up to 95% accuracy. The samples were also analyzed by LIBS-ICP-MS in tandem mode, implicating a depletion of Zn and Ba in samples of SARS-CoV-2 positive subjects that inversely correlate with CN lines in the LIBS spectra.

## Introduction

Almost 2 years after the SARS-CoV-2 virus emerged, the world remains in the grips of the SARS-CoV-2 virus, which is leading countries to take unprecedented action. The current non-pharmaceutical measures that include isolation are taking a high toll on the economy and the health of the world population. To guide health authorities and policy makers, one of the continuously evolving unknowns is the spread of the pathogen in populations on local, regional and national scales. Additionally, with the roll out of vaccines, methods to rapidly determine immune status on an individual basis are required to assess priorities. This measurement technique will also contribute to safe re-opening of society and travel by rapid pre-screening of individuals ahead of conference, public transport or entertainment events.

Widespread serological testing has been used to determine the seroprevalence of populations and regional variations. Angulo et al.^1^ report a seroprevalence in the USA of 14% in mid-November 2020, before the onset of vaccination. Currently, regionally in the USA, estimates are up to 32%^2^. Global national seroprevalence ranges up to 40% (in Kuwait^3^) and require constant updating. Herd immunity may be achieved through combined extensive vaccination and naturally obtained seroprevalence but if successful will likely need to be monitored constantly. This requires rapid testing methods that can be widely implemented. In this contribution we present a feasibility study supporting that Laser Induced Breakdown Spectroscopy (LIBS) combined with machine learning has the potential to be developed into a prime tool to rapidly and accurately identify positive and negative subjects without the need for reagents or extensive training.

### Laser induced breakdown spectroscopy

Laser induced breakdown spectroscopy (LIBS) is an optical technique that employs a pulsed laser to generate a plasma, which vaporises a small portion of a sample^4^. The UV–Visible–NIR light emitted by the plasma is collected and recorded by a spectrometer. This produces a spectrum of emitted light that is characteristic of a sample and can be used as a fingerprint. The detection of the emitted light is time-resolved which allows optimization for atomic, ionic or molecular lines. LIBS is a versatile technique that has been used to obtain compositional information down to the mg/kg concentrations in gases, fluids and solids (for overview see^4^). It owes its recent increase in popularity to this versatility as well as the speed of analysis and relative experimental simplicity requiring next to no sample preparation and its success as the geochemist on Mars Rover, “Curiosity”^5^. LIBS has also been used successfully for human and animal health^6^, including in the detection of biomarkers of cancers, viruses and bacteria^7–12^.

## Methods

### Samples

Ninety-seven human plasma samples were collected in sodium citrate anticoagulant from donors drawn by MRN Diagnostics both before (pre 11/2019) and after the appearance of SARS-CoV-2 in the United States. The samples were collected under the National Expanded Access protocol sponsored by the Mayo Clinic. Informed consent was obtained from all subjects and studies were performed in accordance with FDA guidelines and regulations including IRB approval. Sample collection was approved by the MRN Diagnostics Institutional Review Board.

Fifty samples collected before the pandemic were used as control or SARS-CoV-2 “negative” samples. The remaining 47 samples were collected a minimum of 21 days after a confirmed SARS-CoV-2 PCR result from an EUA approved test and will be referred to here as “positive”. This second sample set had IgG levels ranging between 2.18 and 8.64 index, sig/co. All samples were heat inactivated at 56 °C for 1 h and stored at <  − 20 °C until their analysis. The donor sample set includes a mix of blood types, ages and sex.

The 97 samples provided by MRN Diagnostics were analysed by two groups, at McGill University (full set of 97 samples) and at the University of Massachusetts (subset of 32 positive and 11 negative samples), using different LIBS instruments and different approaches (see below for details). This resulted in three different LIBS datasets from UMass and McGill and one ICP-MS dataset. The data was subsequently shared between labs and analysed by both groups to confirm each other’s results. This approach was chosen to validate the results using different LIBS instruments and different analytical methodology.

### LIBS analysis—UMass

Prior to performing LIBS measurements of the blood plasma samples, 5 μl of each individual blood plasma specimen were deposited on the unpolished side of pure Si wafers. These wafers were previously rinsed in 2-propanol. The blood plasma samples deposited on the Si wafers were then dried for 10 min using a Tungsten infrared lamp. During laser ablation, individual single-shot spectra from adjacent spots of the deposited drop were acquired. The LIBS measurements were performed on a total of 43 samples that consists of 32 positive heat-treated samples and 11 negative heat-treated.

The LIBS experimental setup used for this work is described elsewhere (e.g.^12^). Briefly, it consists of focusing 7 ns Nd:YAG laser (Surelite II, Continuum) pulses operating at 1064 nm on samples using an air-spaced doublet lens with focal length of 30 mm. The blood plasma samples were loaded onto a 3-D computer-controlled translation stage located within a chamber (SciTrace, AtomTrace) and the laser-induced plasma emission collected using a 50 μm core-diameter optical set at an angle of 45° with respect to the laser beam. The optical fiber was coupled to an Echelle spectrograph (Andor Technology, ME 5000) and a thermoelectrically cooled iStar Intensified Charge Coupled Device (ICCD) camera (Andor Technology, DH734-18F-03). The time parameters used for this work were: 1 μs gate delay and a 5 μs gate width. The focused laser spot diameter was about 100 μm, the repetition rate was set at ½ Hz, and the laser energy was 130 ± 2 mJ. All measurements were carried out in air at atmospheric pressure.

For each blood plasma sample, 100 single-shot spectra were acquired. Each spectrum was acquired from a fresh spot on the surface of the dried blood plasma drop by using the 3-D translation stage to displace the blood plasma sample after each laser shot. For the analysis, the 100 single-shot spectra acquired for each sample were averaged, following removal of the outlier spectra i.e., spectra with a total emission intensity outside the interval mean ± 1 standard deviation.

### LIBS-ICP-MS analysis—McGill

Samples were kept in a freezer at − 20 °C at McGill University and thawed shortly before analysis. To minimise the contribution of the sample substrate on the analyses, 0.45 mm PVDF Millipore Sigma Durapore filters were used. These filters contain some Na and K and they were therefore washed in 4% HNO_3_ for 30 min at 80 °C and 30 min in nanopure H_2_O at 80 °C which reduced the Na and K to levels barely detectable by LIBS. Blank filters were also analysed in every session. 20 μl of plasma was pipetted onto a filter and dried under a heat lamp. Not all plasma drops dried in the same way and some formed a rim around a domed surface. Others caused the filters to curl. Most however formed a homogeneous flat crust on top of the filter.

The LIBS, a J200 from Applied Spectra, is directly coupled to a quadrupole ICP-MS, an iCAP Qc from Thermo Finnigan. The LIBS has a Czerny–Turner spectrometer and an ICCD detector (LIBS 2) with gate delay and width control and a 2400 grating, additionally it has 6 channel CCD broadband detectors (LIBS 1) with an electronic pulse delay generator enabling gate delay adjustment. The laser is a 213 nm Nd:YAG laser operated at 10 Hz. The gate delay of both LIBS 1 and 2 was set to 0.05 μs with 1.05 μs and 10 μs gate width for LIBS 1 and 2 respectively. These parameters were optimised for signal to background ratio and maximum information density. The spot size was set to 60 μm, laser energy (1.5 mJ) and scan speed were chosen for optimal ablation of the dried plasma without penetrating the filter. The laser was fired at the samples after purging the chamber and filling it with helium. The LIBS collectors were aimed at the resultant plasma while larger particles were whisked away by the helium flow and transported to the ICP-MS.

Dried blood plasma drops were analysed as line scans of 5 mm length and 10 s duration resulting in 100 individual shots. Each line was treated as a single analysis comprised of 100 accumulated LIBS spectra and 10 s of averaged counts for the ICP-MS. Between 1 and 7 line scans were run for each blood plasma drop as well as a number of duplicate drops to verify that the manner of drying did not affect the spectra. After scrutiny for anomalous spectra, the data from individual lines scans of the sample drop were averaged. Additionally, each day 2–3 blank filters were analysed and a NIST610 glass was analysed to monitor for drift. No systematic drift was observed.

The helium-sample flow (800 ml/min) was mixed with argon (1100 ml/min) upon exiting the chamber and transported to a second Ar plasma in the ICP-MS. All masses were measured sequentially with 20 ms per mass and an overall total analysis time of 10 s. Analysed masses are: ^7^Li, ^9^Be, ^11^B, ^19^F, ^23^Na, ^24^Mg, ^27^Al, ^29^Si, ^31^P, ^35^Cl, ^39^K, ^43^Ca, ^44^Ca, ^45^Sc, ^47^Ti, ^51^V, ^53^Cr, ^55^Mn, ^57^Fe, ^59^Co, ^60^Ni, ^65^Cu, ^66^Zn, ^69^ Ga, ^74^Ge, ^75^As, ^77^Se, ^81^Br, ^85^Rb, ^88^Sr, ^89^Y, ^90^Zr, ^93^Nb, ^95^Mo, ^107^Ag, ^111^Cd, ^115^In, ^118^Sn, ^125^Te, ^133^Cs, ^137^Ba, ^139^La, ^140^Ce, ^141^Pr, ^143^Nd, ^147^Sm, ^153^Eu, ^157^Gd, ^159^Tb, ^163^Dy, ^165^Ho, ^166^Er, ^169^Tm, ^172^Yb, ^178^Hf, ^181^Ta, ^182^W, ^197^Au, ^205^Tl, ^208^Pb, ^209^Bi, ^232^Th, ^238^U. Interference of Se on Ge and Sn on In were automatically corrected for. Of this list, ^9^Be, ^11^B, ^19^F, ^45^Sc, ^53^Cr, ^89^Y, ^93^Nb, ^111^Cd, ^115^In, ^125^Te, ^139^La, ^140^Ce, ^141^Pr, ^143^Nd, ^147^Sm, ^153^Eu, ^157^Gd, ^159^Tb, ^163^Dy, ^165^Ho, ^166^Er, ^169^Tm, ^172^Yb, ^178^Hf, ^181^Ta, ^232^Th, were below the detection limit in > 75% of the samples. ^209^Bi was detected only in the negative samples. ICP-MS data were collected in time-resolved mode and processed using the Iolite software^13^.

## Data processing

### Data description and pre-processing—McGill

The initial data set consists of 97 samples, including 50 negative and 47 positive donors, all three McGill datasets (LIBS1, LIBS2, ICP-MS) passed initial quality checks.

The ICP-MS data was analyzed semi-quantitively and reported as counts per second with the background measured immediately prior to each line scan subtracted. To account for differences in ablation yield, each line was normalised to ^43^Ca and multiplied by the Ca II transition in the LIBS spectrum (393 nm in LIBS1) after the LIBS spectra were normalised to the total intensity and accumulated. The multiplication with the LIBS Ca peak was done to account for any variability in Ca between the samples. Calcium was chosen, as it is an element that can be analysed well between both techniques. This method allows us to observe variability in Ca using LIBS, but not from the ICP-MS. Additionally ^44^Ca was analysed by ICP-MS, however this mass has a potential interference from C and N.

**Figure 1:**
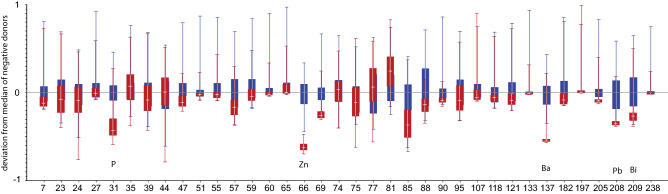
Variance in ICP-MS data between positive (red) and negative (blue) samples. A median mass spectrum of the negative samples has been subtracted from all spectra. All data was scaled to the 5–95 percentile range for each element. Error bars denote the interquartile range.

For the ICP-MS data, only elements for which at least half of the samples were above the detection limit were kept in the dataset. For these elements the values for samples that were below detection limit were set to 2/3 of the minimum value for that element in the dataset. This ensures that these values play no role in the classification while keeping as many variables in the dataset as possible. Single mass outliers, defined as exceeding the 95-percentile level, were set to the median value of the respective positive or negative classes for the same reason, to prevent them from dominating the analysis. A median mass spectrum of all known negative samples was subtracted from every analysis and all elements were scaled to the 5–95 percentile range. Figure 1 shows the scaled variance in the trace elements from ICP-MS of the positive and negative samples.

**Figure 2:**
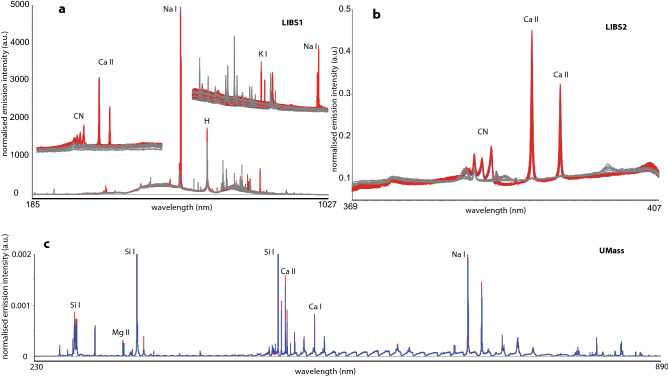
LIBS spectra from LIBS 1, LIBS 2 and UMass. Cumulative and averaged (UMass) spectra of 100 shots. Red are positive samples; blue are negative samples. LIBS 1 and LIBS 2 were deposited on PDVF filters (grey), UMass samples were deposited on Si wafers. All spectra are normalised to total intensity.

The LIBS spectra were normalised to total intensity and accumulated over the line scans. Spectra were visualised and scrutinised for anomalous spectra as a result of focussing issues, which were discarded (Fig. 2). To reduce the number of variables and because the aim was to fuse the LIBS data with the mass spectra from the ICP-MS, features were selected, and their peak heights used rather than the full data spectra. Feature selection was done by hand and verified using PCA to rule out exclusion of peaks with high variance. For the broadband LIBS 1 spectra, 95 features were selected, for the high-resolution LIBS 2, 12 features.

The plasma samples were analysed on PVDF filters. The contribution of these filters to the analyses was identified with the help of the blank spectra and a principal component analysis. Where the substrate contributed strongly to the analysis, these samples were discarded, which affected only a few analyses. The main contributors from the filter substrate were F and Si. F and Si peaks were therefore eliminated from the dataset and line scans with prominent F peaks were discarded. Si and F were similarly omitted from the ICP-MS dataset. We note that the filter contribution seemed to be a larger for the negative samples than for the positive samples.

The ICP data and the selected features from LIBS 1 and 2 datasets were fused since they were obtained simultaneously. To prevent over-representation of one of the fused datasets all datasets were scaled in the same way: to the median value of the negative analyses and scaling to the 5–95 percentile range of each variable. In the following discussion we will distinguish 3 datasets: fused ICP-LIBS1-LIBS2, LIBS1 alone, LIBS2 alone. These sample sets were split in training sets (78) and test sets (19). Each training and test set contains a similar number of positive and negative samples.

### Data description and pre-processing—UMass

The dataset at UMass contains 43 samples out of the same original 97 samples that were analysed at McGill. To analyse the LIBS data, all the spectra belonging to each category (Positive (P) and Negative (N)) were first averaged. Lorentzian profiles were then fit to the emission lines present only in the samples for mean P, N spectra. Figure 2 shows typical mean LIBS spectra obtained.

**Figure 3:**
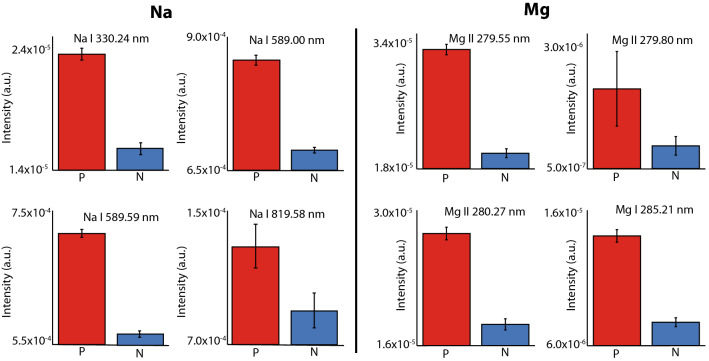
Comparison of the emission intensity of Na I and Mg I–II transitions in positive (red), and negative treated (blue) samples. The error bars represent the fitting error of the average spectra of positive (P) and negative (N).Transitions based on Refs.^14,15^.

A spectroscopic analysis thus obtained based on comparing the intensities of specific emission lines reveals differences to within one standard deviation of the mean between the two classes of plasma blood samples. These differences are shown in Fig. 3. The error bars represent the fitting error of the average spectra of positive and negative.

**Figure 4:**
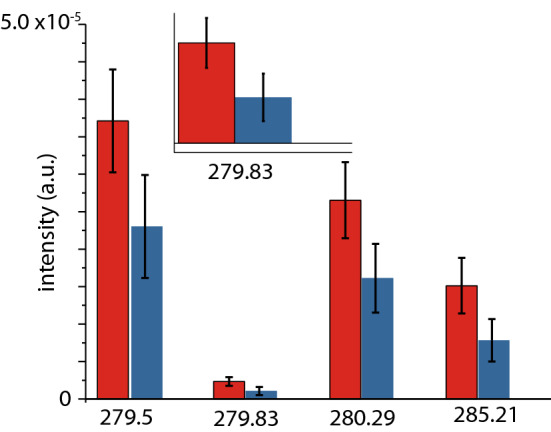
Comparison of the emission intensity of Mg I–II transitions in positive (red), and negative treated (blue) samples. Bars display the average between the fitted emission intensity of LIBS spectra of each individual sample, and the error bar is the standard deviation of the mean.

It is also worth noting that we have observed the same trend for K I. For a more meaningful data treatment, which could also account for the inter-sample differences within each class, the emission intensity of each of the mentioned transitions in the LIBS spectra of each sample (again with a Lorentzian fit) was determined and then averaged. These results are reported in Fig. 4, where the error bars represent the standard deviation of the mean emission intensity of each class. This figure shows that, for Mg I–II transitions, the differences between P and N samples remain significant. For the Na, K, no significant difference was observed between the two classes when using this more conservative data analysis approach.

## Discriminatory analyses

### PCA-LDA

Linear Discriminant Analysis (LDA) is a supervised classification method that defines a linear function to separate different groupings, here plasma from positive and negative donors, in multidimensions. This method has been used to distinguish different oils or minerals base on LIBS spectra (e.g.^16,17^). LDA cannot be applied with large numbers of variables and is prone to model overfitting and is thus often combined with principal component analysis (PCA) to reduce the dimensionality of the database. PCA is an unsupervised technique that is used to derive a new coordinate system combining variables along the directions of maximum variance within the dataset. A challenge of PCA is to limit the number of allowable principal components to describe only the data structure and not the noise. As a first approach we model our datasets using PCA-LDA.

**Figure 5:**
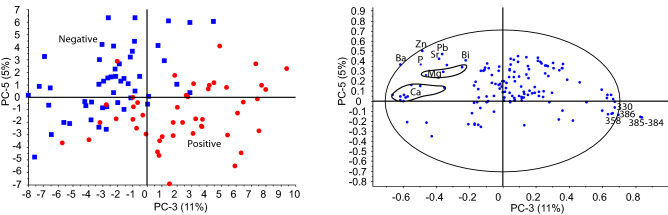
PCA of fused data of all 97 samples. Only PC 3 and 5 are shown here as they distinguish samples from positive and negative donors on the score plot. The loading plot shows some of the elements in all three sub datasets (LIBS 1, LIBS 2, ICP-MS), that distinguish the two clusters. Note Ba, Zn, P, Pb, Sr, Mg are correlated, and are anti-correlated to 385–384, 386, 358 and 330 nm.

To ensure our models are robust we bootstrapped the data by splitting the dataset (97) samples into different training (78) and test sets (19) with similar proportions of positive and negative samples. PCA on each training set was used to cast the data into a different coordinate system and reduce the number of variables. The test sets were subsequently projected into the new coordinate system to derive projected PC’s for the test set. Assignment of variables to PC’s thus differs per training set. As input files for the LDA we used the PCA scores and projected scores for each training and test set and let the LDA algorithm decide which PC’s were significant in the discrimination using a 1% confidence interval. The reason for this approach was the observation that most of the variance in the dataset, PC1, does not pertain to differences in immune response to SARS-CoV-2 (Fig. 5).

This makes sense since our dataset was chosen to encompass a range of blood types, sex and ages. The LDA confirms that later PC’s, i.e. more subtle variations, distinguish positive and negative. The number of PC’s that were used in the LDA differs per training set and the most important (largest F ratio) PC differs as well. However, the loadings of the most important PC’s implicate a restricted set of elements and lines (Table 1). The accuracy, specificity and sensitivity of the models are given in Table 1. These were calculated from the probabilities of false positive and false negative in each of the test sets of 19 samples. They are thus calculated on test sets that were not used in defining the PCA-LDA discrimination model.

**Table1:**
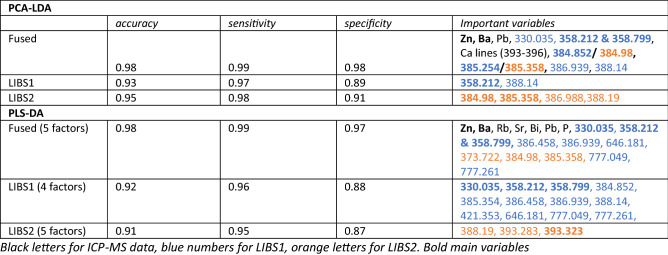
Results of PCA-LDA and PLS-DA.

### PLS-DA

Partial Least Squares Discriminant Analysis has also been successfully applied with spectral data (e.g.^11,18^). Like PCA, PLS-DA combines variables to reduce the dimensionality of the data. Unlike PCA, however PLS-DA is a supervised method that uses the assigned clusters to find the directions of maximum discrimination between the clusters. PLS-DA works well for extracting variables that are important in discriminating clusters.

**Figure 6:**
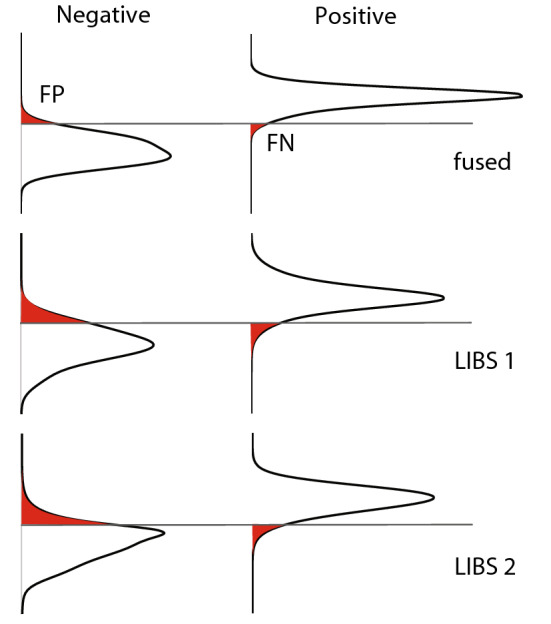
Cumulative probabilities of positive versus negative result in test set of 19 samples.

For the PLS-DA analysis, the Unscrambler^®^ software by Camo with a PLS-R implementation was used but with a hard classification and using the NIPALS algorithm. The robustness of the models was tested by running a cross validation using the entire 97 sample dataset and leaving 6 random samples out every time. For every model, the model Pearson correlation coefficient (r) versus factor was plotted graphically and the optimal number of factors was graphically established at the highest median r and lowest r interquartile range. This point represents where the PLS-DA models are most reproducible and before overfitting increases the spread in performance of the models again. The models were subsequently run with the training set alone (79 samples) and used to predict the classification for the test set (19 samples). Individual probabilities in the test set data were summed to create a cumulative probability (Fig. 6). The cumulative probability was used to calculate the false positive and false negative probabilities. For each model the optimal number of factors, accuracy of the model, and probability of false positive and false negative test results are listed in Table 1. As can be seen in Fig. 6, false negatives are less common than false positives.

## Discussion

### Model performance

All of the models perform well in distinguishing between blood plasma from positive and negative donors (Table 1). Both for the PCA-LDA and the PLS-DA, the fused dataset outperforms the LIBS datasets. Both PCA-LDA and PLS-DA models are able to distinguish the samples from positive and negative donors and implicate the same variable to be important in the distinction, with some differences. The PC analysis shows that the main variability in the data, and hence differences between the donors, may not be caused by an immune response to SARS-CoV-2 but could lie in other factors that were not investigated in this study. This is to be expected as these donors were selected to represent a diverse group in terms of their sex, blood type and age. The power of this machine learning approach is that by using it we were able to isolate an underlying more subtle response caused by a single pathogen, the SARS-CoV-2. The dataset ideally should be scaled up to include even more diversity to rule out any other commonalities within the positive and negative clusters. The ICP-MS data is superior to the LIBS data because it includes trace-elements down to the ppt (ng/kg) range. As opposed to the ppm (mg/kg) for LIBS. However a drawback is that for field applications, ICP-MS data are more costly to obtain and require a technician to perform the analysis. As a rapid test ICP-MS is less ideal, thus ICP-MS was not the focus of this study. However, the combination of ICP-MS and LIBS sheds light on the combinations of major and trace-elements that play a role in the immune response, as discussed below.

Mg I–II lines that are an important distinguishing variable in the UMass dataset do feature in the LIBS 1 dataset but are of relatively low intensity and play a lesser role. The 828.17 nm line observed in the UMass data does not appear in LIBS 1. Possible reasons are differences in the sample substrate and instrument characteristics. Important, however, is that all three LIBS datasets are able to distinguish samples from positive and negative donors with 91% accuracy or more. The success of these three datasets suggests that a robust and accurate distinction can be made regardless of spectrometer.

### Important variables

Both PLS-DA and PCA-LDA indicate a consistent set of variables that distinguish positive and negative samples. These include Zn and Ba from the ICP-MS, lines 358.212, 358.799, 384.852 (384.98 in LIBS 2), 385.254 (385.358 in LIBS 2), 386.939 (386.988 in LIBS 2) and 388.14 nm (388.19 in LIBS 2) (Table 1). Note that there is a calibration shift between LIBS 1 and LIBS 2, LIBS 1 is somewhat mis-calibrated relative to LIBS 2. Zn and Ba, and, to a lesser extent, P, Pb, Rb are correlated and higher in negative samples, and they are anti-correlated with 330.035, 358.212 & 358.799 and 384.98, 385.358, 386.988, 388.19 nm. Lines 358.212 & 358.799 and the lines at 384.98, 385.358, 386.988, 388.19 nm (LIBS 2) could potentially be CN lines^19^. The absence of these lines in the blank filter suggests that these potential CN lines are derived from the samples either direct or by recombination in the plasma.

Differences between PLS-DA and PCA-LDA models result from differences in modelling approaches. Importantly, all models consistently point to important roles for Zn and Ba on one direction and potential CN lines in the other. The UMass dataset implicates Mg, likely because this data was collected on a different instrument under different running conditions resulting in different element sensitivity. Thus, the two datasets acquired in two different laboratories should be seen as complementary. In the UMass dataset Mg lines feature prominently and these are also seen in LIBS 1 albeit at lower intensity and no clear distinction between positive and negative sample could be seen in Mg transitions alone in LIBS 1. Figure 4 shows that Mg concentrations in the UMass subset are higher in the samples from positive donors. Figure 5 suggests that some of the variance in Mg transitions (in LIBS 1) correlates with Zn. The data thus suggest that a multivariate analysis of the data is imperative. In all datasets we also observe differences, to within one standard deviation of the mean, in the level of Na and Ca. In the UMass subset this is indicated by Na-I lines at 330.24, 589.00, 589.59, 819.58 and 330 nm (Fig. 3). In the fused dataset 330.135 nm is an important variable and could be a Na-I line (Table 1). However, none of the higher intensity lines of Na, such as 589.00 nm and 589.59 nm show a clear distinction. This may be caused by self-absorption. The importance of Ca is however clear from LIBS 1 and LIBS 2 by the inclusion of 393.323 nm (393.366 nm) and 396.775 nm (396.847 nm) lines as well as the 373.722 nm (373.69 nm) and the 421.353 nm (422.007 nm) and 646.181 nm (646.256 and 645.687 nm) (Table 1).

There may be multiple underlying processes for lower relative concentration in Zn and Ba and higher relative intensities in potential CN lines in individual samples. However, machine learning is based on the premise that the dataset captures the diversity of plasma samples in the population and extracts only those features that pertain to the factors sought. To better understand what distinguishes the plasma from donors that tested positive from those who did not, a study of the association of elements and their correlation that can implicate a specific (set of) proteins is needed. Ideally such an analysis is done with a larger set of samples. In the current (limited) database Zn and Ba covary (with P, Pb, Sr, Rb, Bi, Mg, Na and possibly Ca) and anticorrelate with potential CN lines. One hypothesis could implicate the destruction of a type of protein whereby the cations are lost.

### A role for Zn and Ba in SARS-CoV-2 response?

The occurrence of Zinc in the list of important variables is of particular interest. Zinc plays an important role in the immune system (e.g.^20^) and has also been linked frequently in relation to SARS-CoV-2. Skalny et al.^21^ provide a review of the role of Zn in the immune system in relation to SARS-CoV-2. Decreased Zn levels are associated with increased susceptibility of inflammatory and infectious diseases and respiratory tract infections including SARS-CoV-2. Experiments have shown that Zn inhibits coronavirus RNA polymerase, inhibiting its ability to replicate^22^. Zinc has also been linked to a decreased activity of the receptor of SARS-CoV-2 (ACE2) (e.g.^21^). Interestingly, a deficiency in Zn has been linked to reduced sense of taste and smell (e.g.^23^), one of the characteristic symptoms of SARS-CoV-2. Low levels of Zn have also been associated with a poor outcome of a SARS-CoV2 infection^24,25^. Our study does not show whether Zn deficiency contributed to a SARS-CoV2 infection or resulted from the infection. It is not known what percentage of the positive donors in our study required hospital treatment. Mayor-Ibarguren et al.^26^ hypothesize that a Zn deficiency could facilitate an infection of SARS-CoV-2 due to an increase in ACE-2 activity. Several studies are currently assesing the role of Zn in treatment of SARS-CoV2 (see^27^ for a review). Heller et al.^28^ analysed serum samples from positive donors including both survivors and non-survivors and noted that over 70% of the non-survivors and 40% of the survivors were deficient in Zn. Our study provides further indication that Zn is an important biomarker with a role in SARS-CoV-2.

Other elements that have been suggested to play a role in SARS-CoV-2 outcome are Se, Cu and Fe (review by Ref.^29^). Heller et al.^28^ noted combined deficiencies in Zn and Se in positive donors. Neither Se nor Cu covary with Zn in our dataset, but Ba and P do. Barium has an unknown biological role, except that it binds to phosphoinositide-specific phospholipase^30^. Phosphorous is an essential element and a component of DNA, RNA, ATP as well as proteins and enzymes. A P deficiency, although our data are not quantitative and cannot be used to indicate a deficiency, can lead to increased risk of infection and confusion as well as muscle weakness and bone defects. All these elements covary and are lower or depleted in samples from positive donors, implicating a common mechanism or protein.

## Summary and conclusion

The world urgently needs new methods to reliably and rapidly detect immunity against SARS-CoV-2 in the population, to guide health authorities and policy makers and prevent new waves of infections. This preliminary study shows that two different LIBS systems with different lasers and different detectors can detect differences between plasma from donors who have previously tested positive for SARS-CoV-2 and plasma donated prior to the emergence of the virus. The results are robust across different detectors, substrates and data analysis methods. LIBS analysis requires minimal sample treatment, no reagents and can be developed into to a fast and reliable instrument when combined with machine learning. Laser Induced Breakdown Spectroscopy thus has the potential to aid in the detection of level of immunity obtained in populations.

In this contribution we have shown that:

- Plasma from positive and negative SARS-CoV-2 donors can be distinguished by LIBS via an elemental fingerprint, which includes elements like Mg, Na, Ca and CN.
- Using machine learning, plasma from positive and negative SARS-CoV-2 donors can be distinguished with up to 95% accuracy and with high sensitivity (98%) and specificity (91%) by LIBS. These data were obtained on three different LIBS spectrographs in two different labs.
- Tandem LIBS-ICP-MS analysis shows that plasma from positive donors is distinguish from that of negative donors by lower Zn and Ba, these elements are anti-correlated with potential CN spectral lines.
- The inclusion of Zn in this list is important to note since Zn has an important role in immune response and is being studied for its role in the treatment of SARS-CoV-2.

Although the dataset presented in this study only contains 97 donors, the results are robust between two different instruments. A larger dataset needs to be analysed in future studies.
